# A Golden Child

**Published:** 1871-12

**Authors:** 


### A GOLDEN CHILD.

On a public occasion a child was coated with gold leaf, and died in a few hours after, because it was not known that the article was impervious to air, and that if the skin was not ventilated, a fatal result would follow.

If a man is encased in India rubber, his sufferings become intolerable in a few hours, and death would follow in a day; hence no garment should be impervious to air.

